# Exploring self-reported health behavior change following naturalistic psychedelic use

**DOI:** 10.1177/13591053251392867

**Published:** 2025-12-31

**Authors:** Laura C. Carvalho, Jorge Encantado, Arlen C. Moller, Talea Cornelius, Natasza Marrouch, Matthew Johnson, Albert Garcia-Romeu, Diogo Veiga, Pedro J. Teixeira

**Affiliations:** 1 University of Lisbon, Cruz-Quebrada-Dafundo, Portugal; 2 Illinois Institute of Technology, Chicago, IL, USA; 3 Columbia University Irving Medical Center, New York, NY, USA; 4 University of Connecticut, Storrs, CT, USA; 5 Sheppard Pratt Center of Excellence in Psilocybin Research and Treatment, Baltimore, MD, USA; 6 Johns Hopkins School of Medicine, Baltimore, MD, USA

**Keywords:** health behavior change, psychedelics, behavioral psychedelics, public health, health

## Abstract

Health-related behaviors are essential determinants of health and well-being, yet unhealthy patterns persist globally. Psychedelics may represent an innovative tool for facilitating positive behavior change. This study investigated retrospective health-related behavior changes attributed to past psychedelic experiences. An online survey was completed by 271 adults who reported on the characteristics of their most impactful psychedelic experience and associated changes across 74 behaviors, including physical activity, and time spent in nature. The most frequently reported changes were in contemplative practices (63%), time spent in nature (55%), and personally meaningful social activities (54%). The majority of changes were reported in a healthy direction. Higher ratings of perceived meaningfulness and having a set intention emerged as significantly associated with overall healthy change. While causality cannot be inferred, these findings highlight the potential of psychedelic experiences as catalysts for health-related behavior change and underscore the role of intention and perceived meaningfulness as candidate mechanisms.

## Introduction

Non-communicable diseases (NCDs), including cardiovascular diseases, cancer, and diabetes, were responsible for 75% of all deaths in 2021– approximately 43 million people – and are the leading cause of premature mortality, with 18 million deaths occurring before the age of 70 (World Health Organization [WHO], 2024a). Health-related behaviors, such as physical inactivity, unhealthy diet, and tobacco and alcohol misuse are key modifiable risk factors for NCDs (WHO, 2024a). Global trends are worrisome: 1.4 billion adults remain physically inactive (WHO, 2022), and 43% of adults are overweight, with 16% living with obesity (WHO, 2024b). While adopting a healthy lifestyle is central to preventing NCDs, decades of theory-based intervention efforts have struggled to promote behavioral change that is both initiated and sustained across the lifespan (Hagger et al., 2022).

In this context, Teixeira et al. (2022) argued that psychedelic experiences could be a novel avenue to support individuals in adopting healthier behaviors. Both clinical and observational studies suggest psychedelics (or psychedelic-assisted therapy) may be effective in treating tobacco (Daldegan-Bueno et al., 2022; Johnson et al., 2014, 2017a, 2017b; Jones et al., 2022) and alcohol misuse (Bogenschutz et al., 2015, 2022; Garcia-Romeu et al., 2019; Perkins et al., 2022). In some of these studies, participants also reported spontaneous improvements in health behaviors not directly targeted by the intervention, such as increased physical activity and healthier eating patterns (Daldegan-Bueno et al., 2022; Garcia-Romeu et al., 2019, 2020; Johnson et al., 2017a). Additional observational studies investigating the impact of naturalistic psychedelic use – that is, occurring in “real life,” outside the clinical and research settings – reinforce the potential of psychedelic experiences for health behavior change, with participants indicating improvements in diet and physical activity as a consequence of psychedelic use (Aday et al., 2024; Bathje et al., 2024; Garcia-Romeu et al., 2020; Lowe et al., 2024; Raison et al., 2022).

Several studies exploring the association between psychedelic use and physical health also suggest an association with a positive lifestyle. Individuals reporting lifetime classic psychedelic use had lower odds of hypertension (Simonsson et al., 2021a), heart disease, diabetes (Simonsson et al., 2021b), of being overweight or obese, and reported better overall health (Simonsson et al., 2021c). Further, psychological insight during one’s most meaningful psychedelic experience has been associated with healthier exercise habits and greater likelihood of maintaining a healthy body mass index (Simonsson et al., 2022). From a public health perspective, a large-scale study of ayahuasca users in Spain found that, compared with the Spanish general population, participants reported higher consumption of fruits and vegetables, higher engagement in yoga or meditation practices, and 55% reported being as physically active as they wished (Ona et al., 2019). This study was replicated in the Netherlands, with Dutch ayahuasca users reporting higher levels of moderate-intensity exercise, more consumption of fruits and legumes, and a higher percentage of participants with a normal BMI (18.5–24.9 kg/m^2^) compared to the general population (Kohek et al., 2023).

Recently, the concept of “behavioral psychedelics” has been proposed as “the study of psychedelics to foster intentional changes in habits and behavior to improve health and resilience” (Neuhaus and Slavich, 2022), reinforcing the importance of considering behavioral patterns and their influence on mental and physical health in studies involving psychedelics. At the population level, the potential of psychedelics to address major public health concerns has led to a new area named Psychedelic Public Health, proposing that the reach of psychedelic studies is extended beyond mental health to include alcohol and tobacco use and chronic disease prevention (Kuiper et al., 2024).

### Purpose

The primary aim of this study was to investigate changes in health-related behaviors reported by psychedelic users following experiences with both classic serotonergic psychedelics (e.g. psilocybin, ayahuasca, DMT, mescaline, LSD) and atypical psychedelic substances (e.g. ibogaine, ketamine, MDMA). The direction (i.e. increase or decrease) and degree of those changes were also assessed, and associations were examined between behavior changes and key characteristics of the psychedelic experience.

## Method

This study employed a cross-sectional design, using a one-time online survey to collect retrospective self-reports of perceived health behavior change following previous psychedelic use. The survey was hosted on the secure Qualtrics platform. A convenience sample of participants was recruited through a study webpage disseminated via social media and university communication platforms. Upon receiving comprehensive study information, participants were invited to complete a one-time survey developed by the research team. The study did not involve the administration of any psychedelic substances or clinical interventions. Instead, it focused exclusively on self-reported perceptions of change following prior psychedelic use in naturalistic, non-controlled settings.

Inclusion criteria were having consumed a psychedelic substance at least once in their lifetime, having a sufficiently good understanding of the English language, and being 18 years or older.

The study received ethical approval from the ethics committee of the Faculty of Human Kinetics, Lisbon University (reference number 17/2023).

### Measures

#### Demographic information

Demographic information collected included age, sex, gender, education level, and employment status. Regarding lifetime drug use, participants were asked about the number of psychedelic experiences, the psychedelics substances ever used, and the psychedelic substance they used most frequently.

#### Self-reports of health behavior change and degree of change

First, participants were presented with a list of health-related behaviors and asked to select those in which they experienced a change, related to a past psychedelic experience. The list included multidimensional health-related behaviors^1^ – physical activity, diet and nutrition, eating patterns/relation with food, contemplative practices, time spent in nature, personally meaningful social activities, and work-life balance – and unidimensional behaviors – alcohol consumption, caffeine consumption, tobacco use, cannabis use, other (non-psychedelic) drug use, psychiatric medication use, non-prescribed medication use, sleep, compliance with public health recommendations, screen use, ice/cold bath and sweat lodge/sauna use. Whenever multidimensional health-related behaviors were selected (e.g. physical activity), participants were presented with a list of specific behaviors (e.g. walking) and asked to select those in which they experienced a change. The following specific behaviors where presented to participants: (1) physical activity: walking, running, swimming, cycling, hiking, physical exercise, yoga, pilates, dancing, martial arts, active mobility, team sports; (2) diet and nutrition: consumption of fruits and vegetables, consumption of vegan or vegetarian meals, consumption of all meats, consumption of red meat, consumption of fish, consumption of dairy products, consumption of animal product alternatives, consumption of legumes, consumption of sugar-based foods and drinks, consumption of processed foods, consumption of nuts and whole grains; (3) eating patterns: eating local/seasonal/organic foods, slow mindful eating, eating according to one’s body needs, food choices according to environmental concerns, food choices according to health concerns, food choices according to animal welfare, binges or cravings, ability to balance calories in versus calories out, flexible eating, enjoyment of a broader range of foods without guilt, purgative behaviors; (4) contemplative practices: meditation, mindfulness, prayer, spiritual practices, stress reduction practices; (5) time spent in nature: gardening, in urban greenspaces, in surrounding greenspaces, in urban blue spaces, in surrounding blue spaces, of “high quality” time spent in nature; (6) personally meaningful social activities: volunteering, community engagement, joining a group, social gatherings, political participation, religious services and events; (7) work-life balance: scheduling limits, reducing time at work/working, not working during leisure hours, family time.

After this initial identification of unidimensional and specific behaviors where a change was experienced, participants were asked to select a maximum of three (e.g. alcohol consumption, walking, and sleep) in which the changes experienced were most impactful (“top three behaviors”), and rate the degree of change on a five-point scale from “increased a lot” to “decreased a lot.” The midpoint of the scale represented mixed changes, capturing cases where changes occurred but not in a consistent direction – that is, instances involving both increases and decreases in the behavior. This category was designed to reflect the dynamic and often non-linear nature of behavior change, distinguishing these patterns from complete stability (“no change”) while acknowledging that the overall direction of change was not uniform.

#### Characteristics of the psychedelic experience

For each “top three behavior,” participants were presented with a series of questions designed to assess the characteristics of the psychedelic experience that most influenced the change: substance used (classic psychedelics, ketamine, MDMA, ibogaine), dose (standard/high dose or low dose), setting of the experience (e.g. ceremonial setting, outdoors in nature), the presence of a guide/facilitator, somatic activities (e.g. breathwork, yoga), preparation practices (e.g. therapy, dietary changes), having set an intention for the experience, and the degree of meaningfulness of the experience (Griffiths et al., 2006). See Supplemental File_1 for more detailed information.

### Data analysis

Changes in health-related behaviors were summarized using descriptive analyses. Frequencies and percentages were calculated to show the proportion of participants who reported changes in each behavior, as well as the degree of those changes.

To investigate which characteristics of the psychedelic experience (i.e. intention, meaningfulness, presence of a guide, preparation practices, and setting) significantly predicted overall changes in health-related behaviors, we conducted multilevel modeling (MLM) with random intercept to account for multiple behaviors nested within participants. The outcome was the degree of change in each behavior, scaled such that −2 represented “decreased a lot,” to +2 represented “increased a lot,” with higher scores indicating greater positive change (0 indicated “mixed” change). Behaviors with fewer than 10 reports – non-prescribed medication use, compliance with public health recommendations, ice/cold bath, and sweat lodge/sauna use – were excluded. Additional unidimensional behaviors were also excluded due to the difficulty in determining their health-related valence. For example, when it comes to psychiatric medication, describing increases in their use as negative may exclude cases of increases in compliance with treatment, which is a positive outcome. The behaviors excluded were psychiatric medication use, cannabis use, caffeine consumption, and other drug use. For a similar reason, based on World Health Organization Guidelines (World Health Organization, Food and Agriculture Organization of the United Nations, & United Nations Children’s Fund, 2024), certain specific behaviors were excluded within diet and nutrition – consumption of vegan/vegetarian meals, consumption of red meat, consumption of animal products alternatives, consumption of all meat, consumption of dairy products – and within eating patterns – food choices according to environmental concerns, food choices according to animal welfare. Behaviors included in this analysis are available at Supplemental File_2.

Descriptive analyses were conducted using the IBM SPSS 29.0 statistical software, and multilevel model analysis were conducted in R using “lme4” and “lmerTest” packages.

## Results

### Descriptive statistics

#### Responded characteristics

The final sample included 271 participants, and demographic information was provided by 100 participants (demographic questions were optional), with a mean age of 41.94 years (SD = 13.96). Just over half the sample identified as female (54%), 85% reported having obtained a college degree, and nearly half (43%) reported being employed full-time. Regarding lifetime psychedelic use participants reported an average of 30.8 experiences (SD = 97.48; Md = 20), and psilocybin mushrooms were the most commonly reported psychedelic substance used (72%). More detailed information is available in Table 1.

#### Characteristics of the most significant psychedelic experience

The majority of reports indicated the use of a standard/high dose of psychedelics (89.7%), with psilocybin mushrooms being the most commonly used substance (41.6%), followed by ayahuasca (15.8%) and LSD (13.1%). Ibogaine was not reported by any participants, while mescaline (1.5%) and ketamine (1.2%) were the least reported substances. The most frequently reported settings of use were “at home or private non-ceremonial” (39%), “in a ceremonial setting” (29%), and “outdoors in nature in a non-ceremonial setting” (12.7%). Few participants reported other settings, with “in a public space” (3.2%) and “at a party” (2.5%) being the least common. The presence of a guide or facilitator was reported in 48.9% of cases, and 59.5% of reports indicated the presence of others during the experience, primarily friends (38.9%), strangers (30.6%), romantic partners (23.6%), and family members (7.2%).

For somatic activities, the most reported activity was meditation (25.5%), followed by none (22.2%) and breathwork (18.1%). Qigong (3.6%) and Taichi (1.3%) were the least reported bodily-based activities engaged during the psychedelic experiences.

Regarding preparation practices, 19.6% of reports indicated mindfulness or meditation practices as part of their preparation, while 18.7% of participants indicated that they did not engage in any preparation practices. Many participants either did not set a specific intention for behavior change (45.9%) or did not set an intention at all for the psychedelic experience (27.6%). A large proportion of participants considered the experience among the 5–10 most meaningful in their life (46.3%).

#### Self-reports of health-behavior change

The health-related behaviors with more than half the participants reporting a change were contemplative practices (62.7%), time spent in nature (55%) and personally meaningful social activities (53.9), followed by work-life balance (43.9%) and alcohol consumption (40.6%). The same behaviors were the most frequently identified by participants as their most impactful behavior changes (“top three” behavior changes): contemplative practices (45.4%), personally meaningful social activities (30.6%), time spent in nature (26.9%), alcohol consumption (21.8%) and work-life balance (21.4%). Conversely, the behaviors least frequently reported were compliance with public health recommendations (8.1%), other (non-psychedelic) drug use (7.7%), sweat lodge/sauna use (7.4%) and non-prescribed medication use (4.1%). Similarly, the behaviors least frequently identified as their most impactful changes were caffeine consumption (1.8%), non-prescribed medication use (1.1%), and compliance with public health recommendations (1.1%).

Within the multidimensional health behavior physical activity, the most impactful changes (“top three” behavior changes) were reported in physical exercise (37.9%), walking (34.5%), yoga (31%), and hiking (31%). Regarding diet and nutrition, the specific behaviors with the most impactful changes included the consumption of vegetables and fruits (37.5%), processed foods (37.5%), and sugar-based foods and drinks (34.4%). For the multidimensional health-related behavior eating patterns, slow mindful eating (44.4%), eating according to one’s body needs (38.9%), eating according to health concerns, and flexible eating (both 22.2%) were indicated as the specific behaviors with most impactful changes. The most impactful changes within contemplative practices were the specific behaviors mindfulness (67.5%) and meditation (45.5%). Changes in time spent in nature were primarily related to the specific behavior high-quality time (45.2%). Concerning personally meaningful social activities, the specific behaviors social gatherings (34.9%) and community engagement (25.3%) were most commonly identified as the most impactful changes. Finally, within the multidimensional health-related behavior work-life balance, the specific behaviors scheduling limits and family time were reported as the most impactful changes, each by 32.8% of participants.

Figures 1–8 display the percentage of participants who identified each behavior as their most significant changes (top three behaviors). A comprehensive breakdown of self-reported behavior changes is provided in Tables 1 and 2 of Supplemental File 3.

**Figure 1.**
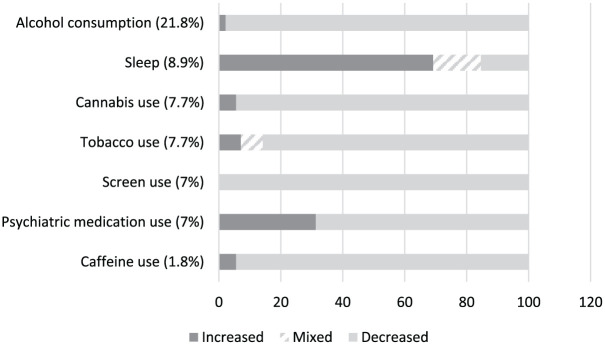
Unidimensional behaviors.

**Figure 2.**
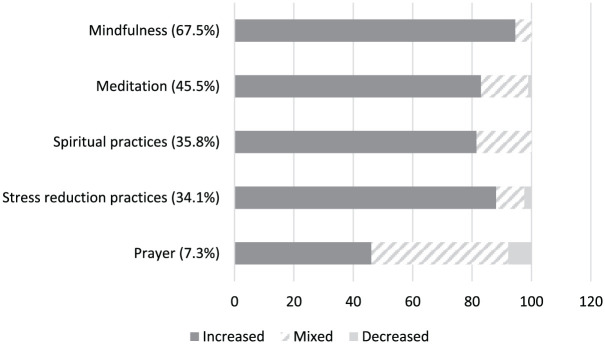
Contemplative practices (45.4%).

**Figure 3.**
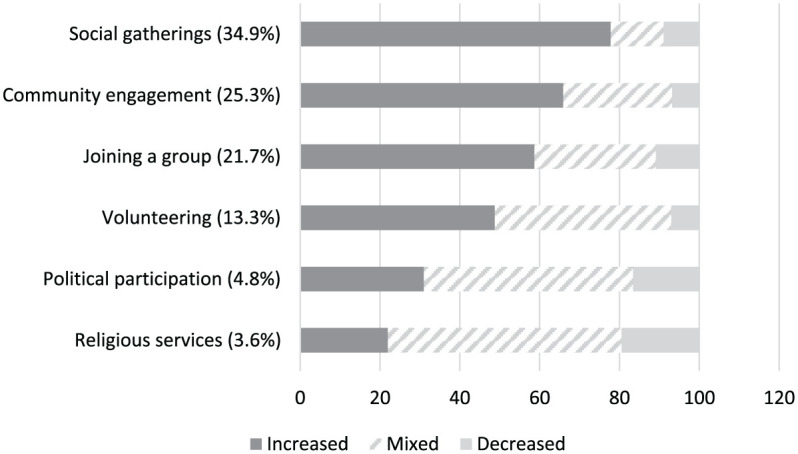
Personally meaningful social activities (30.6%).

**Figure 4.**
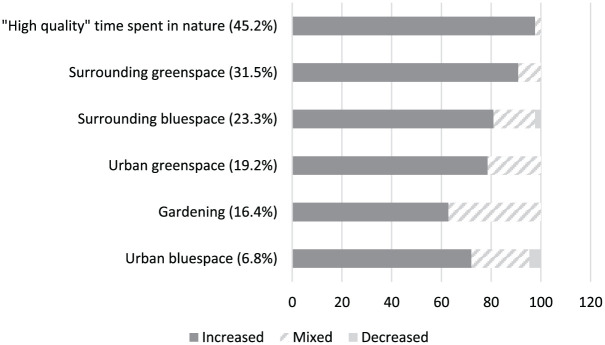
Time spent in nature (26.9%).

**Figure 5.**
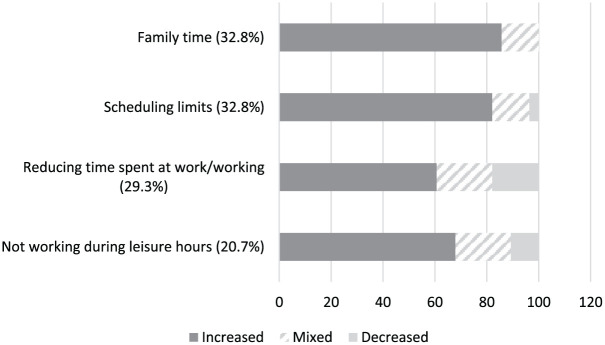
Work-life balance (21.4%).

**Figure 6.**
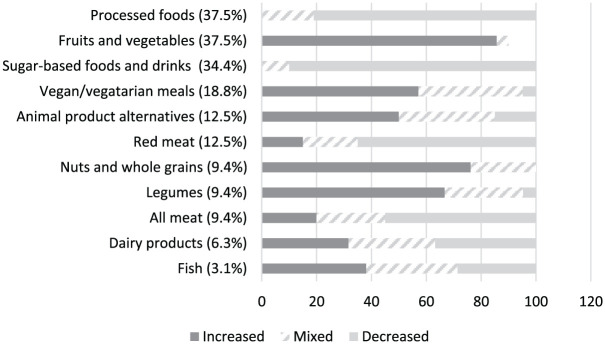
Diet and nutrition (11.8%).

**Figure 7.**
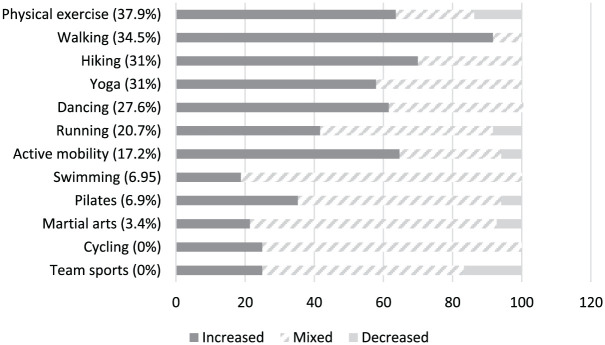
Physical activity (10.7%).

**Figure 8.**
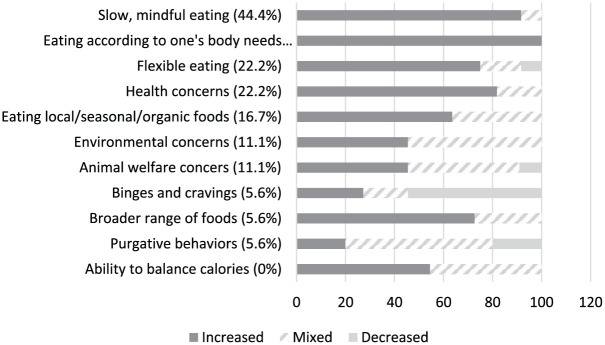
Eating patterns (6.6%).

**Table 1.** Demographic and lifetime psychedelic use (*n* = 100).

Demographic characteristic	% or Mean (SD)
Mean age (SD)	41.9 (13.96)
Female (gender)	54
Education	
Less than high school	1
Graduated high school	5
Trade/technical school	2
Some college (no degree)	7
Bachelor’s degree	27
Master’s degree	34
Doctorate/professional degree	24
Employment status	
Full-time (40 hours)	43
Part-time (39 hours)	6
Unemployed	5
Student	13
Retired	9
Homemaker	1
Self-employed	20
Unable to work	3
Mean lifetime psychedelic use (SD)	30.8 (97.48)
Psychedelics used in the past (most often used)	
Psilocybin mushrooms	72 (39)
Psilocybin truffles	22 (8)
Ayahuasca	21 (9)
DMT (not ayahuasca)	22 (1)
Peyote/San Pedro/synthetic mescaline	15 (2)
LSD	44 (14)
MDMA	47 (19)
Ketamine	20 (2)
Ibogaine	0
Combination of substances	20 (2)
Other	18 (4)

SD: standard deviation.

**Table 2.** Associations between characteristics of the psychedelic experience overall changes in behavior.

Term	Estimate	Std. error	*t*	*p*-Value
(Intercept)	0.550067855	0.27159493	2.02532450	0.044
**Intention**	**0.233306616**	**0.08896118**	**2.62256658**	**0.009**
**Meaningfulness**	**0.110015814**	**0.04036876**	**2.72527116**	**0.007**
Guide	0.037860933	0.08634738	0.43847228	0.661
Preparation	−0.007212265	0.08578328	−0.08407542	0.933
Setting	0.039449981	0.02793221	1.41234747	0.159

Bold text indicates statistically significant associations.

#### Direction of behavior changes

Figures 1–8 display the percentages of reported increases and decreases for all behaviors analyzed. Table 3 of Supplemental File 3 provides detailed information on the percentages reported. Given the small number of reports in the degree of change (less than 10), the health-related behaviors non-prescribed medication use, compliance with public health recommendations, ice/cold bath, and sweat lodge/sauna use, were not included in this analysis.

Regarding unidimensional behaviors, decreases were predominantly reported for alcohol consumption (97.8% of those who changed this behavior), caffeine consumption (94.4%), cannabis use (94.4%), tobacco use (85.7%), medically prescribed psychiatric medication use (68.8%), and screen use (100%). Additionally, most participants reported increases in sleep quality (69.2%).

For multidimensional behaviors, the degree of change was reported for each specific behavior where a change was experienced. The majority of reports indicated increases across all specific time spent in nature activities (e.g. of “high quality” time spent in nature: 97.7%) and work-life balance (e.g. family time: 85.7%), while among contemplative practices, increases were predominant in all activities (e.g. mindfulness: 94.6%) except prayer, which showed an equal frequency of increased and mixed changes (46.1%).

Regarding physical activities, most participants who reported changes indicated increases (e.g. walking: 91.7%) or mixed changes (e.g. swimming: 81.3). Similarly, for personally meaningful social activities, increases were predominant for four activities (e.g. social gatherings: 77.8%), while two showed a majority of mixed changes (e.g. religious services: 58.5%).

Within diet and nutrition, five options showed a majority of increases (e.g. consumption of vegetables and fruits: 85.7%), while daaecreases were predominant for four options (e.g. consumption of sugar-based foods and drinks: 90%).

Finally, regarding eating patterns, increases were mostly reported for six specific eating patterns (e.g. slow mindful eating: 91.7%) and decreases were more common for one specific eating pattern (binge cravings: 54.5%). Food choices based on animal welfare showed equal frequencies of increases and mixed changes (45.5%), while food choices based on environmental concerns (54.5%) and purgative behaviors (60%) showed a higher frequency of mixed changes.

### Associations between characteristics of the psychedelic experience and health behavior change

Results for an intercept-only model revealed a significant overall trend toward healthier changes across all behaviors (β = 0.988, SE = 0.037, *p* ⩽ 0.001). In this model, the variance of the random intercept was 0.154 (SD = 0.392), the residual variance was 0.747 (SD = 0.865), and the intraclass correlation coefficient was 0.171.

Next, a model including all characteristics of the psychedelic experience as fixed effects showed that both the degree of meaningfulness and intention were significantly associated with greater positive behavior change (β = 0.110, SE = 0.040, *p* = 0.007; β = 0.233, SE = 0.089, *p* = 0.009). This suggests that greater perceived meaningfulness and having set a clear intention for the psychedelic experience were linked to more positive behavior changes (Table 2). In this model, the variance of the random intercept was 0.143 (SD = 0.378), the residual variance was 0.454 (SD = 0.674), and the ICC increased to 0.239.

## Discussion

The investigation of health behavior changes in relation to psychedelic use remains largely underexplored. This study’s primary aim was to examine health-related behavior changes attributed to past naturalistic psychedelic experiences, as well as the direction and degree of those changes. Findings indicate that for many participants, psychedelic use was perceived as having contributed to changes in several important health-related behaviors, with the majority of these changes described in a healthier direction.

The behaviors most frequently identified as the most relevant changes (i.e. reported to be among top three behaviors) included increased contemplative practices, engagement in personally meaningful social activities, time spent in nature, and reduced alcohol consumption.

Positive changes were reported across all contemplative practices (e.g. meditation, mindfulness), with the exception of prayer. Previous studies have similarly found that classic psychedelic use is associated with a greater likelihood of practicing mindfulness meditation and with an increased number of days engaging in such practices (Simonsson et al., 2023, 2024). In turn, mindfulness meditation has been shown to be positively associated with both physical and psychological well-being (Jazaieri and Shapiro, 2017).

In the current study, more than half of the participants (53.6%) who reported a change in alcohol consumption identified it as one of the most relevant behavior changes attributed to their psychedelic experience, with the vast majority reporting a reduction in consumption (97.8%). These findings are consistent with both clinical and observational studies suggesting a potential role for psychedelics in reducing alcohol consumption (Bogenschutz et al., 2022; Garcia-Romeu et al., 2019; Kervadec et al., 2024; Teixeira et al., 2025). Given promising outcomes from psychedelic-assisted psychotherapy for alcohol use disorder and converging evidence from naturalistic use, psychedelics may represent a promising avenue for the treatment of alcohol use disorder (Lodetti et al., 2024). Nonetheless, recent findings from a randomized clinical trial found that a single dose of psilocybin combined with psychotherapy was insufficient to effectively prevent relapse in alcohol use disorder (Rieser et al., 2025). However, participants in the psilocybin group reported significant improvements in depressive symptoms, hopelessness, negative affect, and emotion suppression, as well as in quality of life, compared to the placebo group.

Although increased time spent in nature does not necessarily equate to greater subjective nature connectedness (Nisbet and Zelenski, 2013), the reported increases in time spent in nature in our sample may reflect heightened levels of nature relatedness, a construct that has previously been associated with psychedelic use and with enhanced well-being (Aday et al., 2025; Forstmann et al., 2023; Kettner et al., 2019; Longo et al., 2023; Nour et al., 2017). In parallel, increased social connectedness – defined as a greater sense of connection to others – has also been frequently reported following psychedelic use (Kettner et al., 2021; Watts et al., 2017). Fifty-seven percent of our sample reported changes in personally meaningful social activities among their most significant behavior changes, with increases reported in specific activities such as joining a group, social gatherings, and volunteering. These types of changes were similarly described by participants in follow-up interviews from a psilocybin-assisted tobacco cessation trial (Noorani et al., 2018). Both social connection and nature-based interventions (e.g. green exercise) have been found to positively influence mental, cognitive, and physical health outcomes (Holt-Lunstad, 2024; Nejade et al., 2022).

It is noteworthy that the behaviors most frequently reported as having changed more positively relate to one’s relationship to oneself (e.g. contemplative practices, reduced alcohol consumption), others (e.g. personally meaningful social activities), and the environment (e.g. time spent in nature), which may indicate a broader sense of connectedness, as suggested by Watts et al. (2022).

Results also indicate a shift toward a more active lifestyle. Physical activity, commonly defined as any bodily movement produced by skeletal muscles that requires energy expenditure (WHO, 2024b), can be increased not only through formal exercise but also through nature-based and social activities. In fact, physical activity is among the 10 behaviors with the most reported changes (*n* = 91), with walking and hiking reported as the most relevant changes. When health is understood not only in biological terms but also as lived experience, the reported behavioral changes align with the WHO concept of human functioning. Along with morbidity and mortality, human functioning is the third health indicator, incorporating both biological health and the individual experience of being able to act and interact in the world (Bickenbach et al., 2023). Psychedelic experiences may positively influence lived health by encouraging individuals to engage in a broader range of meaningful activities, ultimately promoting a more active, salutogenic, and socially engaged lifestyle. Should further research continue to support the health-promoting potential of psychedelics, it may hold potential not only at the individual level but also as a tool for population-level health promotion (Kuiper et al., 2024). This perspective positions psychedelics not only as therapeutic agents but also as innovative tools for prevention, particularly in the context of chronic disease. By influencing behaviors linked to physical activity, diet, substance use, and psychological well-being, psychedelics may contribute to addressing some of the most pressing public health challenges of our time: non-communicable diseases (NCDs), mental health disorders, and the global burden of lifestyle-related health decline.

Despite a general trend toward healthier changes, some participants reported mixed changes – that is, both increases and decreases in the behavior – and, in some cases, changes not aligned with improved health. For example, high rates of mixed change were reported in some domains, including physical activities such as swimming (83%), eating behaviors such as purgative practices (60%), contemplative practices such as prayer (46%), and social activities such as religious services (59%). Mixed changes may reflect the complex and non-linear nature of behavior change, also suggesting that sustained improvements may require a more structured and intentional integration process. It is also important to acknowledge that not all reported changes were in a healthier direction. A small proportion of participants reported increases in alcohol consumption (2%) and tobacco use (7%), as well as decreases in certain physical activities (e.g. team sports; 17%) and nutritional behaviors (e.g. consumption of legumes; 5%). Although it is possible that some of these decreases could conceivably be healthy for a subset of individuals (e.g. those with a legume allergy, or those who engage in hazardous team sports), these findings nonetheless underscore the need for caution in assuming that psychedelic experiences are uniformly health-promoting and suggest that, in some cases, behavioral changes may reflect shifts in personal priorities or lifestyle disruptions that do not necessarily enhance health. Additionally, given that the maintenance of the behavior change was not assessed, we are limited in our understanding of how psychedelic experiences impact the initiation and maintenance of behaviors.

A recent study by Teixeira et al. (2025) identified similar trends in health-related behavior change, particularly concerning diet, alcohol consumption, and tobacco use. Nearly half of the participants reported positive changes in their diet (49.4%) and tobacco use (48.5%), while 66% reported reductions in alcohol consumption. However, the authors also noted that for a small subset of participants, psychedelic use was associated with negative changes in these behaviors, specifically 2.2% for diet, 3.2% for tobacco use, and 8.3% for alcohol consumption.

The present study also examined the characteristics of the psychedelic experience that most influenced relevant changes (i.e. the top three behaviors). While sample size constraints limit conclusions about specific associations between psychedelic experience characteristics and individual behavior domains, general trends can be observed. Classic psychedelics and high doses were more frequently reported, with non-ceremonial settings (different from a party) collectively more commonly indicated. Naturalistic use of psychedelics is becoming more prevalent around the world (United Nations Office on Drugs and Crime, 2023), which seems to be reflected in the variety of settings reported for psychedelic use in the present sample.

It is also worth noticing that while meditation or mindfulness practices, as well as time spent in nature, were the most commonly reported preparation practices, they were not among the characteristics of the psychedelic experience significantly associated with overall change. Rather, our data shows that higher rates of meaningfulness of the experience and having set an intention were significantly associated with positive changes in behavior. Previous studies have similarly shown that perceived meaningfulness is associated with reductions in tobacco and alcohol use following psychedelic experiences (Garcia-Romeu et al., 2019; Johnson et al., 2017b). While the role of intention remains underexplored, the concept of “set” – referring to an individual’s internal mindset – has long been recognized as shaping the outcome of psychedelic experiences (Hartogsohn, 2017). In a study by Haijen et al. (2018), setting clear intentions was positively associated with the occurrence of mystical-type experiences, which were, in turn, linked to greater well-being post-experience. The “behavioral psychedelics” model proposed by Neuhaus and Slavich (2022), similarly emphasizes the inclusion of intention-setting and behavioral goal development in the preparatory phase as key to fostering intentional, health-promoting behavior change. Although the mechanisms underlying psychedelic-related change remain incompletely understood, evidence points to neural disruptions that enhance psychological flexibility and the capacity to break entrenched patterns (Carhart-Harris et al., 2014; Carhart-Harris and Friston, 2019). Research on alcohol and tobacco (mis)use also identifies improvements in self-efficacy (Bogenschutz et al., 2015; Garcia-Romeu et al., 2019; Jensen et al., 2025; Johnson et al., 2017b), motivation to change (Bogenschutz et al., 2015; Nielson et al., 2018), and improvements in emotional regulation (Agin-Liebes et al., 2024) as relevant psychological factors. Furthermore, both the intensity and personal meaningfulness of the acute mystical-type experience have been linked to sustained reductions or cessation of alcohol and tobacco use (Garcia-Romeu et al., 2019; Johnson et al., 2017b; Nielson et al., 2018).

While the present findings contribute to the growing literature on psychedelic experiences and health-related behavior change, several limitations should be noted. As an observational, non-experimental study, causal inferences cannot be established. Additionally, the self-selected nature of the sample may have introduced selection bias, with individuals having more favorable or meaningful experiences more likely to participate, thus potentially inflating reports of positive outcomes. The relatively small sample size, particularly concerning demographic variables, limited the overall statistical power and precluded meaningful subgroup analyses, thereby reducing the generalizability of the findings. Attribution bias is also a concern as participants may overattribute behavioral changes to the psychedelic experience while overlooking other contributing factors. Finally, reliance on retrospective self-report introduces limitations in measurement accuracy, as behavioral changes were not assessed through objective or validated instruments, as well as the possibility of recall bias regarding the details of the psychedelic experience.

## Conclusion

Findings from the present study support previous results from secondary analysis in clinical and naturalistic research studies demonstrating an association between psychedelic experiences and positive changes in health-related behaviors for some individuals. This study adds novel insights by identifying with greater precision which specific behaviors tend to change most frequently, the relative magnitude of those changes, and the characteristics of psychedelic experiences that may influence them. While causality cannot be established, this can have relevant implications for public health and preventive care, especially given the burden of non-communicable diseases linked to lifestyle factors. Our results provide a foundation for future longitudinal and interventional research exploring how intentional, meaningful psychedelic experiences are situated within the context of factors promoting or thwarting health and health behaviors in the population. Future research should also focus on examining the possible psychosocial mechanisms implicated in behavioral change.

## Supplemental Material

sj-docx-1-hpq-10.1177_13591053251392867 – Supplemental material for Exploring self-reported health behavior change following naturalistic psychedelic use Supplemental material, sj-docx-1-hpq-10.1177_13591053251392867 for Exploring self-reported health behavior change following naturalistic psychedelic use by Laura C. Carvalho, Jorge Encantado, Arlen C. Moller, Talea Cornelius, Natasza Marrouch, Matthew Johnson, Albert Garcia-Romeu, Diogo Veiga and Pedro J. Teixeira in Journal of Health Psychology

sj-docx-2-hpq-10.1177_13591053251392867 – Supplemental material for Exploring self-reported health behavior change following naturalistic psychedelic use Supplemental material, sj-docx-2-hpq-10.1177_13591053251392867 for Exploring self-reported health behavior change following naturalistic psychedelic use by Laura C. Carvalho, Jorge Encantado, Arlen C. Moller, Talea Cornelius, Natasza Marrouch, Matthew Johnson, Albert Garcia-Romeu, Diogo Veiga and Pedro J. Teixeira in Journal of Health Psychology

sj-docx-3-hpq-10.1177_13591053251392867 – Supplemental material for Exploring self-reported health behavior change following naturalistic psychedelic use Supplemental material, sj-docx-3-hpq-10.1177_13591053251392867 for Exploring self-reported health behavior change following naturalistic psychedelic use by Laura C. Carvalho, Jorge Encantado, Arlen C. Moller, Talea Cornelius, Natasza Marrouch, Matthew Johnson, Albert Garcia-Romeu, Diogo Veiga and Pedro J. Teixeira in Journal of Health Psychology

## Notes

1. By “multidimensional health-related behaviors,” we refer to broader categories of behaviors that can be broken down into specific, observable actions. For example, physical activity includes specific behaviors such as walking, running, and dancing.
