# Supplementary material for: Exploring self-reported health behavior change following naturalistic psychedelic use
Source: J Health Psychol. 2025 Dec 31;31(9):3651–67. doi: 10.1177/13591053251392867 (PMC13365305; doi:10.1177/13591053251392867)
Supplement: sj-docx-1-hpq-10.1177_13591053251392867 – Supplemental material for Exploring self-reported health behavior change following naturalistic psychedelic use [file sj-docx-1-hpq-10.1177_13591053251392867.docx]

**Supplementary file 2**

*Behaviors categories and sub-behaviors for LLM analysis*

**Single behaviors**

- Alcohol consumption *- Reverse coded*
- Tobacco use - *Reverse coded*
- Screen use - *Reverse coded*
- Sleep quality

**Sub-behaviors (physical activity)**

- Physical exercise
- Walking
- Yoga
- Hiking
- Dancing
- Running
- Active mobility
- Swimming
- Pilates
- Martial Arts
- Cycling
- Team sports

**Sub-behaviors (within diet and nutrition)**

- Consumption of vegetables and fruits
- Consumption of processed foods - *Reverse coded*
- Consumption of Sugar-based foods / drinks - *Reverse coded*
- Consumption of legumes
- Consumption of nuts and whole grains
- Consumption of fish

**Sub-behaviors (within eating patterns)**

- Slow, mindful eating
- Eating according to body needs
- Flexible eating
- Binges and cravings - *Reverse coded*
- Ability to balance calories
- Enjoyment of a broader range of foods
- Purgative behaviors - *Reverse coded*
- Eating local/seasonal/organic foods
- Food choices according to health concerns

**Sub-behaviors (within contemplative practices)**

- Mindfulness
- Meditation
- Stress reduction practices
- Spiritual practices
- Prayer

**Sub-behaviors (within time spent in nature)**

- High quality time spent in nature
- Surrounding greenspace
- Surrounding bluespace
- Urban greenspace
- Gardening
- Urban bluespace

**Sub-behaviors (within social activities)**

- Social gatherings
- Community engagement
- Joining a group
- Volunteering
- Political participation
- Religious services

**Sub-behaviors (within work-life balance)**

- Scheduling limits
- Family time
- Reducing time spent working
- Not working leisure hours

**Single behaviors NOT included:**

- Psychiatric medication
- Cannabis use
- Caffeine consumption
- Non-prescribed medication use
- Other drug use (e.g., cocaine, opioids)
- Compliance with public health recommendations
- Ice / cold shower or bath
- Sauna use

**Subcategories of behaviors NOT included:**

Within diet and nutrition:

- Consumption of vegan / vegetarian meals
- Consumption of read meat
- Consumption of animal products alternatives
- Consumption of all meat
- Consumption of dairy products

Within eating patterns

- Food choices according to environmental concerns
- Food choices according to animal welfare concerns
