# Supplementary material for: Exploring self-reported health behavior change following naturalistic psychedelic use
Source: J Health Psychol. 2025 Dec 31;31(9):3651–67. doi: 10.1177/13591053251392867 (PMC13365305; doi:10.1177/13591053251392867)
Supplement: sj-docx-2-hpq-10.1177_13591053251392867 – Supplemental material for Exploring self-reported health behavior change following naturalistic psychedelic use [file sj-docx-2-hpq-10.1177_13591053251392867.docx]

**Supplementary file 1**

**Characteristics of the psychedelic experience** (adapted from Johnson et al., 2017)

1. **Substance used**

Psilocybin mushrooms

Psilocybin truffles

Ayahuasca

DMT (not ayahuasca)

Peyote / San Pedro / synthetic mescaline

LSD

MDMA

Ketamine

Ibogaine

Combination of substances

Other

1. **Dose**

Standard / high dose

Low dose (lower that the standard dose)

1. **Setting of the experience**

In a ceremonial setting (e.g., retreat, church, private session, in nature, etc)

At home / private non-ceremonial (different from a party)

Outdoors in nature in a non-ceremonial setting (different from a party)

At a party

At a concert or festival

In a public space (e.g., shopping mall, movie theater, etc)

In a research setting (e.g., hospital, university, etc)

Other

1. **Presence of a guide / facilitator**

Yes

No

1. **Presence of other participants**

Yes

No

1. **Other participants**

Family

Romantic partner

Friends

Strangers

1. **Somatic activities**

Breathwork

Yoga

Meditation practices

Dancing

Qigong

Tai-Chi

Other

None

1. **Preparation practices**

Therapy

Dietary changes (not including traditional “dietas”)

Physical activity

Mindfulness / meditation practices

Journaling

Contact with nature

Other

None

1. **Intention to change a specific health behavior?**

Yes

No

I did not set an intention for this experience

1. **Degree of meaningfulness of the experience** (Griffiths et al., 2006).

The single most meaningful experience of my life

Among the 5/10 most meaningful experiences of my life

Similar to a meaningful experience that occur on average once every 5 years

Similar to a meaningful experience that occur on average once a year

Similar to a meaningful experience that occur regularly (monthly / weekly)

No more than routine, everyday experiences

Not meaningful
