# Supplementary material for: Exploring self-reported health behavior change following naturalistic psychedelic use
Source: J Health Psychol. 2025 Dec 31;31(9):3651–67. doi: 10.1177/13591053251392867 (PMC13365305; doi:10.1177/13591053251392867)
Supplement: sj-docx-3-hpq-10.1177_13591053251392867 – Supplemental material for Exploring self-reported health behavior change following naturalistic psychedelic use [file sj-docx-3-hpq-10.1177_13591053251392867.docx]

**Supplementary File 3**

**Table 1**

*Self-reported changes in health behaviors (n = 271)*

| Total of self-reported changes | | “Top 3 behaviors” (i.e., most significant behavior changes) |
| --- | --- | --- |
| Behavior | % Change | % Change |
| Contemplative practices | 62.7% | 45.4% |
| Time spent in nature | 55% | 26.9% |
| Social activities | 53.9% | 30.6% |
| Work-life balance | 43.9% | 21.4% |
| Alcohol consumption | 40.6% | 21.8% |
| Diet and nutrition | 33.9% | 11.8% |
| Physical activity | 33.6% | 10.7% |
| Eating patterns | 31% | 6.6% |
| Sleep | 29.9% | 8.9% |
| Cannabis use | 21.4% | 7.7% |
| Screen use | 21.4% | 7% |
| Tobacco use | 19.9% | 7.7% |
| Caffeine consumption | 14.8% | 1.8% |
| Ice/cold bath or shower | 14.8% | 2.6% |
| Psychiatric med. | 12.5% | 7% |
| Compliance with public health recommendations | 8.1% | 1.1% |
| Other drug use | 7.7% | 2.2% |
| Sweat lodge/sauna use | 7.4% | 0 |
| Non-prescribed medication use | 4.1% | 1.1% |

**Table 2**

*Self-reported changes in specific behaviors within multidimensional behaviors*

| Total of self-reported changes | | “Top 3 behaviors” (i.e., most significant behavior changes) |
| --- | --- | --- |
| Behavior | % Change (% Change total sample) | % Change |
| Contemplative practices (n = 123) | | |
| Mindfulness | 75.6% (34.3%) | 67.5% |
| Meditation | 71.5% (32.5%) | 45.5% |
| Stress reduction practices | 68.3% (31%) | 34.1% |
| Other (e.g., art practice) | 66.7% (30.3%) | 19.5% |
| Spiritual practices | 65.9% (29.9%) | 35.8% |
| Prayer | 61.8% (28%) | 7.3% |
| Personally meaningful social activities (n = 83) | | |
| Other (e.g., family gatherings) | 57.8% (17.7%) | 38.6% |
| Joining a group | 55.4% (17%) | 21.7% |
| Social gatherings | 54.2% (16.6%) | 34.9% |
| Community engagement | 53% (16.2%) | 25.3% |
| Volunteering | 51.8% (15.9%) | 13.3% |
| Political participation | 50.6% (15.5%) | 4.8% |
| Religious services | 49.4% (15.1%) | 3.6% |
| Time spent in nature (n = 73) | | |
| Surrounding greenspace | 60.3% (16.2%) | 31.5% |
| High quality time | 60.3% (16.2%) | 45.2% |
| Gardening | 58.9% (15.9%) | 16.4% |
| Urban bluespace | 58.9% (15.9%) | 6.8% |
| Urban greenspace | 57.5% (15.5%) | 19.2% |
| Surrounding bluespace | 57.5% (15.5%) | 23.3% |
| Other (e.g., forest bathing) | 56.2% (15.1%) | 45.2% |
| Work-life balance (n = 58) | | |
| Scheduling limits | 48.3% (10.3%) | 32.8% |
| Reducing time | 48.3% (10.3%) | 29.3% |
| Not working leisure hours | 48.3% (10.3%) | 20.7% |
| Family time | 48.3% (10.3%) | 32.8% |
| Other (e.g., more quality time at work) | 48.3% (10.3%) | 17.2% |
| Diet and nutrition (n = 32) | | |
| Fruits and vegetables | 65.6% (7.7%) | 37.5% |
| Vegan/vegetarian meals | 65.6% (7.7%) | 18.8% |
| Fish | 65.6% (7.7%) | 3.1% |
| Legumes | 65.6% (7.7%) | 9.4% |
| Processed foods | 65.6% (7.7%) | 37.5% |
| Nuts and whole grains | 65.6% (7.7%) | 9.4% |
| All meats | 62.5% (7.4%) | 9.4% |
| Red meat | 62.5% (7.4%) | 12.5% |
| Animal product alternatives | 62.5% (7.4%) | 12.5% |
| Sugar-based foods/drinks | 62.5% (7.4%) | 34.4% |
| Dairy products | 59.4% (7%) | 6.3% |
| Other | 56.3% (6.6%) | 3.1% |
| Physical activity (n = 29) | | |
| Walking | 82.8% (8.9%) | 34.5% |
| Running | 82.8% (8.9%) | 20.7% |
| Physical exercise | 75.9% (8.1%) | 37.9% |
| Hiking | 69% (7.4%) | 31% |
| Yoga | 65.5% (7%) | 31% |
| Dancing | 62.1% (6.6%) | 27.6% |
| Pilates | 58.6% (6.3%) | 6.9% |
| Active mobility | 58.6% (6.3%) | 17.2% |
| Swimming | 55.2% (5.9%) | 6.9% |
| Cycling | 55.2% (5.9%) | 0% |
| Martial arts | 48.3% (5.2%) | 3.4% |
| Other (e.g., snowboarding) | 48.3% (5.2%) | 20.7% |
| Team sports | 41.4% (4.4%) | 0% |
| Eating patterns (n = 18) | | |
| Slow, mindful eating | 66.7% (4.4%) | 44.4% |
| Body needs | 66.7% (4.4%) | 38.9% |
| Flexible eating | 66.7% (4.4%) | 22.2% |
| Eating local | 61.1% (4.1%) | 16.7% |
| Environmental concerns | 61.1% (4.1%) | 11.1% |
| Health concerns | 61.1% (4.1%) | 22.2% |
| Animal welfare concerns | 61.1% (4.1%) | 11.1% |
| Binges cravings | 61.1% (4.1%) | 5.6% |
| Ability balance calories | 61.1% (4.1%) | 0% |
| Broader range of foods | 61.1% (4.1%) | 5.6% |
| Other | 61.1% (4.1%) | 0 |
| Purgative behaviors | 55.6% (3.7%) | 5.6% |

**Table 3**

*Direction of health behavior changes*

| Health behaviors | | Increased_(a)_ | Mixed | Decreased_(b)_ |
| --- | --- | --- | --- | --- |
|  |  | % | % | % |
|  | Screen use (n = 12) | 0 | 0 | 100% |
|  | Alcohol consumption (n = 45) | 2.2% | 0 | 97.8% |
|  | Caffeine consumption (n = 18) | 5.6% | 0 | 94.4% |
|  | Cannabis use (n = 18) | 5.6% | 0 | 94.4% |
|  | Tobacco use (n = 14) | 7.1% | 7.1% | 85.7% |
|  | Psychiatric medication use  (n = 16) | 31.3% | 0 | 68.8% |
|  | Sleep (n = 13) | 69.2% | 15.4% | 15.4% |
| *Physical activity* | Walking (n = 24) | 91.7% | 8.3% | 0 |
|  | Hiking (n = 20) | 70% | 30% | 0 |
|  | Active mobility (n = 17) | 64.7% | 29.4% | 5.9% |
|  | Physical exercise (n = 22) | 63.6% | 22.7% | 13.6% |
|  | Dancing (n = 18) | 61.1% | 38.9% | 0 |
|  | Yoga (n = 19) | 57.9% | 42.1% | 0 |
|  | Running (n = 24) | 41.7% | 50% | 8.3% |
|  | Pilates (n = 17) | 35.3% | 58.8% | 5.9% |
|  | Team sports (n = 12) | 25% | 58.3% | 16.7% |
|  | Cycling (n = 16) | 25% | 75% | 0 |
|  | Martial arts (n = 14) | 21.4% | 71.4% | 7.1% |
|  | Swimming (n = 16) | 18.8% | 81.3% | 0 |
| *Diet and nutrition* | Consumption of sugar-based foods and drinks (n = 20) | 0 | 10% | 90% |
|  | Consumption of vegetables and fruits (n = 21) | 85.7% | 4.3% | 0 |
|  | Consumption of processed foods (n = 21) | 0 | 19% | 81% |
|  | Consumption of nuts and whole grains (n = 21) | 76.2% | 23.8% | 0 |
|  | Consumption of legumes (n = 21) | 66.7% | 28.6% | 4.8% |
|  | Consumption of red meat (n = 20) | 15% | 20% | 65% |
|  | Consumption of vegan/vegetarian meals (n = 21) | 57.1% | 38.1% | 4.8% |
|  | Consumption of all meat (n = 20) | 20% | 25% | 55% |
|  | Consumption of animal product alternatives (n = 20) | 50% | 35% | 15% |
|  | Consumption of fish (n = 21) | 38.1% | 33.3% | 28.6% |
|  | Consumption of dairy products (n = 19) | 31.6% | 31.6% | 36.8% |
| *Eating patterns* | Eating according to one’s body needs (n = 12) | 100% | 0 | 0 |
|  | Slow, mindful eating (n = 12) | 91.7% | 8.3% | 0 |
|  | Eating according to health concerns (n = 11) | 81.8% | 18.2% | 0 |
|  | Flexible eating (n = 12) | 75% | 16.7% | 8.3% |
|  | Enjoyment of a broader range of foods without guilt (n = 11) | 72.7% | 27.3% | 0 |
|  | Eating local/seasonal/organic foods (n = 11) | 63.6% | 36.4% | 0 |
|  | Ability to balance calories in vs calories out (n = 11) | 54.5% | 45.5% | 0 |
|  | Binges and cravings (n = 11) | 27.3% | 18.2% | 54.5% |
|  | Food choices according to environmental concerns (n = 11) | 45.5% | 54.5% | 0 |
|  | Eating according to animal welfare concerns (n = 11) | 45.5% | 45.5% | 9.1% |
|  | Purgative behaviors (n = 10) | 20% | 60% | 20% |
| *Contemplative practices* | Mindfulness (n = 93) | 94.6% | 5.4% | 0 |
|  | Meditation (n = 88) | 83% | 15.9% | 1.1% |
|  | Stress reduction practices (n = 84) | 88.1% | 9.5% | 2.4% |
|  | Spiritual practices (n = 81) | 81.5% | 18.5% | 0 |
|  | Prayer (n = 76) | 46.1% | 46.1% | 7.9% |
| *Time spent in nature* | Of “high quality” time spent in nature (n = 44) | 97.7% | 2.3% | 0 |
|  | In surrounding greenspace (n = 44) | 90.9% | 9.1% | 0 |
|  | In surrounding bluespace (n = 42) | 81% | 16.7% | 2.4% |
|  | In urban greenspace (n = 42) | 78.6% | 21.4% | 0 |
|  | In urban bluespace (n = 43) | 72.1% | 23.3% | 4.7% |
|  | Gardening (n = 43) | 62.8% | 37.2% | 0 |
| *Personally meaningful social activities* | Social gatherings (n = 45) | 77.8% | 13.3% | 8.9% |
|  | Community engagement (n = 44) | 65.9% | 27.3% | 6.8% |
|  | Joining a group (n = 46) | 58.7% | 30.4% | 10.9% |
|  | Volunteering (n = 43) | 48.8% | 44.2% | 7% |
|  | Political participation (n = 42) | 31% | 52.4% | 16.7% |
|  | Religious services (n = 41) | 22% | 58.5% | 19.5% |
| *Work-life balance* | Family time (n = 28) | 85.7% | 14.3% | 0 |
|  | Scheduling limits (n = 28) | 82.1% | 14.3% | 3.6% |
|  | Reducing time spent at work/working (n = 28) | 60.7% | 21.4% | 17.9% |
|  | Not working during leisure hours (n = 28) | 67.9% | 21.4% | 10.7% |

(a) Total of *increased a lot* and *increased a little* responses

(b) Total of *decreased a lot* and *decreased a little* responses
